# Identification of plant cells in black pigments of prehistoric Spanish Levantine rock art by means of a multi-analytical approach. A new method for social identity materialization using *chaîne opératoire*

**DOI:** 10.1371/journal.pone.0172225

**Published:** 2017-02-16

**Authors:** Esther López-Montalvo, Clodoaldo Roldán, Ernestina Badal, Sonia Murcia-Mascarós, Valentín Villaverde

**Affiliations:** 1 UMR 5608 TRACES, French National Center for the Scientific Research (CNRS)-University of Toulouse 2-Jean Jaurès. 5 allées Antonio Machado. Toulouse, France; 2 Material Science Institute of the University of Valencia (ICMUV). C/ Catedrático José Beltrán, 2. Paterna-Valencia, Spain; 3 Department of Prehistory and Archaeology of the University of Valencia (Spain). Avenida de Blasco Ibáñez 28. Valencia, Spain; Max Planck Institute for the Science of Human History, GERMANY

## Abstract

We present a new multi-analytical approach to the characterization of black pigments in Spanish Levantine rock art. This new protocol seeks to identify the raw materials that were used, as well as reconstruct the different technical gestures and decision-making processes involved in the obtaining of these black pigments. For the first of these goals, the pictorial matter of the black figurative motifs documented at the Les Dogues rock art shelter (Ares del Maestre, Castellón, Spain) was characterized through the combination of physicochemical and archeobotanical analyses. During the first stage of our research protocol, *in situ* and non-destructive analyses were carried out by means of portable Energy Dispersive X-Ray Fluorescence spectrometry (EDXRF); during the second stage, samples were analyzed by Optical Microscopy (OM), Raman spectroscopy, and Scanning Electron Microscopy coupled with Energy Dispersive X-ray spectroscopy (SEM-EDX). Two major conclusions have been drawn from these analyses: first, charred plant matter has been identified as a main component of these prehistoric black pigments; and second, angiosperm and conifer charcoal was a primary raw material for pigment production, identified by means of the archaeobotanical study of plant cells. For the second goal, black charcoal pigments were replicated in the laboratory by using different raw materials and binders and by reproducing two main *chaînes opératoires*. The comparative study of the structure and preservation of plant tissues of both prehistoric and experimental pigments by means of SEM-EDX underlines both a complex preparation process and the use of likely pigment recipes, mixing raw material with fatty or oily binders. Finally, the formal and stylistic analysis of the motifs portrayed at Les Dogues allowed us to explore the relationship between identified stylistic phases and black charcoal pigment use, raising new archaeological questions concerning the acquisition of know-how and the transfer of traditionally learned *chaînes opératoires* in Spanish Levantine rock art.

## Introduction

Spanish Levantine rock art is a unique form of pictorial expression in European prehistory. Located in rock shelters in the inland regions of the Iberian Mediterranean basin, this prehistoric art is of particular interest due to both the naturalism of figures and the narrative component of its scenes, which portray specific economic and social activities. The remarkable variations in form, technique, themes, and composition over time and throughout the territory that exist within this graphic horizon underline the interaction between complex social dynamics and a highly structured territory, as manifested through interaction and exchange networks that likely experienced several phases of expansion and contraction through time. This implies that the analysis of Levantine rock art is particularly useful for gaining insight into the prehistoric societies responsible for their elaboration.

The cultural and chronological framework of Levantine rock art is currently imprecise, resulting mainly from the fact that the primarily mineral-based pigments that are used are impossible to date using conventional radiocarbon methods. Nevertheless, the latest chrono-cultural hypotheses support an affiliation between Neolithic societies and these graphic representations [1–2]. Recently, using both archaeological and graphic lines of evidence [3], some scholars have called into question the association between this horizon of pictorial expression and the initial Neolithisation process of the Iberian Mediterranean Basin (6^th^ millennium cal. BC), and rather relate it to the fully established Neolithic societies (5^th^ millennium cal. BC).

### The Levantine rock art palette: State of the debate and novel research perspectives

The pigment palette of Spanish Levantine rock art consists of just three colours: red, black and white, with a clear prevalence of the first throughout the entire relative chronological sequence as well as in most of the Levantine regions. To date little is known regarding the composition, preparation and use of these prehistoric pigments, and subsequently even less is known regarding chronological and geographical variations in these hypothetical *chaînes opératoires*.

Regarding colour selection, we have yet to identify the motivations behind colour choice, whether they correspond to specific social rules or rather are a function of limited access to mineral or organic raw materials. Some regional patterns can be established via differential use of certain colours, yet this argument cannot be pushed further as to date a geographical analysis of spatial distributions of differential colour use and boundary variation through time has not been completed. Regarding raw materials, no geological survey allowing for the geographic reconstruction of possible colouring material sources in the areas surrounding Levantine rock art sites has been conducted.

From a more technical standpoint, data available concerning components used in the preparation of pigments is scarce, and we are far from being able to establish social patterns via the identification of recurring pigment mixtures or “recipes”. Although the physicochemical characterization of Spanish Levantine pigments has been a primary research focus in recent years, the theoretical scope, methods used, and experimental techniques explored are extremely heterogeneous, thereby hampering any sort of interregional comparative study of results produced [4–6]. Investigations have primarily been focused on the characterization of raw materials, while study into technical processes and the decision making involved in the elaboration of pigments have both taken a back seat. These more traditional approaches to pigment study are justified by the assumption that Levantine pigments are the result of simple solutions rather than complex mixtures [7], since components other than minerals, such as proteins or lipids that could act as binders or solvents in mixtures have yet to be detected. However, and as will be demonstrated in this paper, we believe that the absence of such solvents or binders in pigments is more likely due to the limits of the experimental techniques used for their identification and not the actual reflection of Spanish Levantine rock art’s previously assumed simplified technical dimension.

There remains therefore considerable work to be done regarding both the improvement of physicochemical identification protocols and the study of Levantine pigment *chaînes opératoires*. These *chaînes opératoires*–modalities through which raw material is acquired, transformed, and used–correspond to social rules that were learned, shared, and accepted by a community, and therefore should be considered as identity-revealing [8] as they can contribute to the characterization of prehistoric communities, their social boundaries, and interaction networks. Their study should thusly be considered as a powerful tool for building interpretational bridges between social and technical aspects [9–13].

We have developed a new research protocol that combines physicochemical techniques, archeobotanical analysis, and experimental archaeology, within the conceptual framework of *chaîne opératoire*, which takes the first steps towards filling the above-mentioned gaps in recent research. Our primary goal is to reveal the cultural patterns linked to pigment use through a thorough characterization of both raw materials and the sequential technical operations that transformed these materials into pigments. For this purpose, the Spanish Levantine shelter of Les Dogues (Ares del Maestre, Castellón, Spain) was taken as a case study.

### Les Dogues rock art shelter

**Fig 1:**
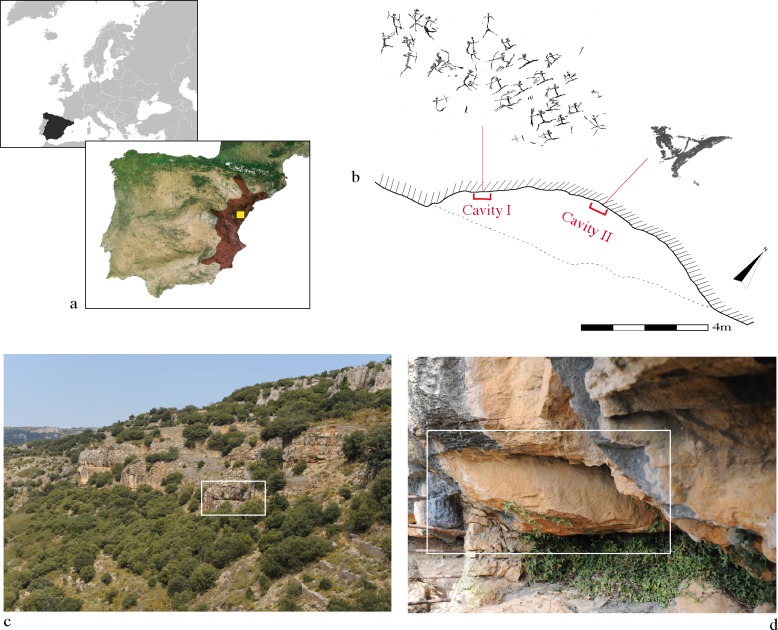
Les Dogues rock art shelter. a. Geographic location of the Les Dogues shelter (Ares del Maestre, Castellón, Spain). The geographic zone of influence of Spanish Levantine rock art is shown in red. b. Floor plan of Les Dogues showing the location of paintings. c. Les Dogues is indicated by the white rectangle. d. Cavity I. Location of the battle scene indicated by the white rectangle. J.B. Porcar’s tracing reprinted from Archivo de Prehistoria Levantina under a CC BY license, with permission from the Editor-in-chief, original copyright 1953.

The Les Dogues rock art shelter (Ares del Maestre, Castellón, Spain) is found in the Gassulla area, one of the largest regions of prehistoric art, in which 14 rock art shelters with Levantine and Iberian Schematic paintings have been documented to date (Fig 1A).

**Fig 2:**
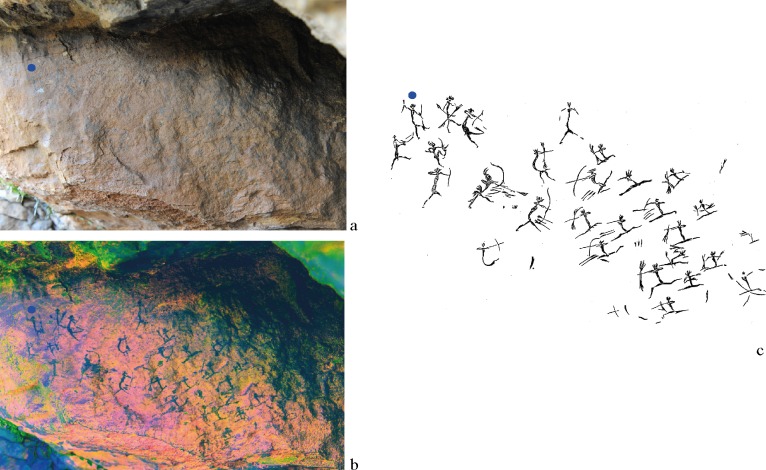
Battle scene of Les Dogues located in cavity I. a. Image showing the low visibility of motifs to the naked eye. b. Image enhancement tools (D-Stretch for Image J) augment the visibility of the poorly preserved charcoal pigments and facilitate the accurate reading of motifs. c. Tracing by J.B. Porcar (1953). Reprinted from Archivo de Prehistoria Levantina under a CC BY license, with permission from the Editor-in-chief, original copyright 1953.

Les Dogues is a shallow rock shelter barely 10 m long, in which two cavities can be distinguished (Fig 1B and 1C). Cavity I contains a battle scene involving up to 29 human figures (Fig 1D), while only 5 human motifs have been preserved in cavity II, located on the lower part of the ledge that protects the shelter. All the motifs portrayed belong to the Spanish Levantine graphic horizon. Even though Les Dogues is not one of the most relevant rock art shelters in the area, in terms of number of depictions and decorative stylistic phases, it is a rather exceptional site as it contains one of the few well-preserved battle scenes known within the Spanish Levantine graphic horizon [14–16] (Fig 2). Furthermore, from a technical point of view, Les Dogues is the only site in the Valltorta-Gassulla area in which all motifs, corresponding to two different Levantine styles, were painted in black.

Today most of these motifs are practically invisible to the naked eye (Fig 2A), this being the result of degradation from exposition to direct sunlight, as well as other atmospheric and bacterial agents, which have also favoured fading and the formation of the associated surface patina [17].

## Materials and methods

The multi-analytical characterization of black pigments from Les Dogues was a multi-step process. Firstly, non-destructive analyses were carried out by means of Energy Dispersive X-Ray Fluorescence spectrometry (EDXRF). Secondly, four samples were analyzed by Optical Microscopy (OM), Raman Spectroscopy (RS) and Scanning Electron Microscopy coupled with Energy Dispersive X-ray spectroscopy (SEM-EDX); all of these methods are well-established analytical tools for the study of parietal and portable rock art [6, 18–22]. Thirdly, an archeobotanical study of samples was carried out in order to determine plant species. Finally, in the hopes of better understanding charcoal pigment *chaînes opératoires*, two main sequences were replicated in laboratory conditions. Samples of both replicated and prehistoric pigments were observed with SEM-EDX to conduct a comparative analysis of the structure, anatomy, and state of preservation of plant tissues.

The General Directorate of Cultural Heritage of the Government of Valencia gave us permission to study the pigments from Les Dogues.

### EDXRF

**Fig 3:**
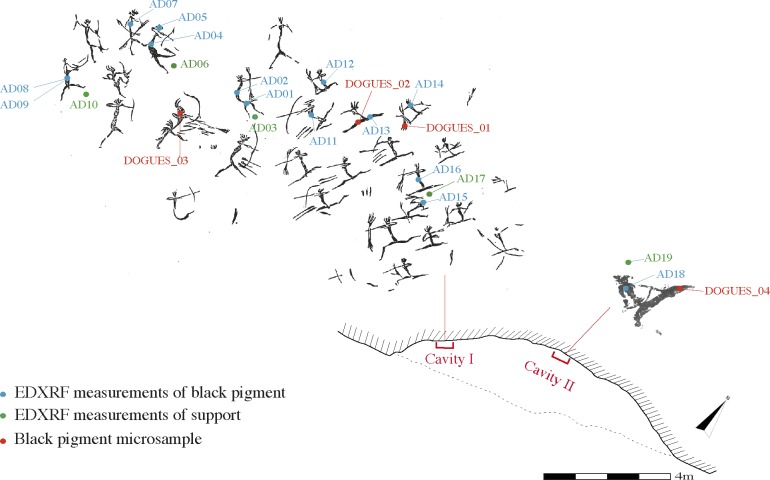
Localisation of the *in situ* measurement points and the four motifs sampled. J.B Porcar’s tracing reprinted from Archivo de Prehistoria Levantina under a CC BY license, with permission from the Editor-in-chief, original copyright 1953.

Non-invasive *in situ* analyses were performed by means of a portable X-Ray Fluorescence spectrometer. In total 14 points were measured, these being selected from 10 motifs in cavity I and 1 motif in cavity II; 5 points from the underlying rock surface were also analyzed for comparative purposes (Fig 3).

**Fig 4:**
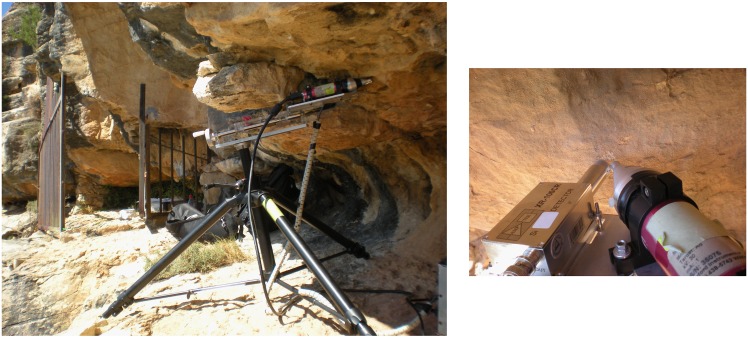
Portable EDXRF spectrometer used in Les Dogues.

The EDXRF analyses were carried out using a portable spectrometer equipped with: 1) a small X-ray tube with a silver anode operating in transmission mode (V_max_ = 30 kV, I_max_ = 0.1 mA), emitting a continuous flow of X-rays with a 3 mm diameter aluminium collimator; 2) a Si-PIN thermoelectrically cooled semiconductor detector, with an energy resolution of 170 eV (FWHM@5.9 keV); 3) a multichannel buffer board MCA Pocket 8000 A; and 4) a mechanical device fitted with a tripod allowing for movements on a XYZ axis to define and optimise the geometry of measurements and ensure their reproducibility (Fig 4).

The X-ray tube and detector were orientated at a 45° angle and the beam and sample were perpendicular to one another. The sample-detector distance was controlled by means of laser diodes and fixed to 1.5 cm in order to increase the geometric efficiency of the system. The EDXRF spectra were registered with an acquisition time of 200 s, a fixed voltage of 30 kV, and a current of 0.004 mA. These parameters are appropriate to excite and detect the fluorescence lines from a wide range of elements with an acceptable statistical significance. The EDXRF spectra were analyzed with the PyMCA software package [23], and fluorescence line areas were normalized to the total spectra count to reduce geometrical effects and fluctuations associated with the X-ray tube during the measurements. The portability of the spectrometer, as well as the non-destructive and reliable nature of EDXRF spectrometry make it an extremely useful tool for *in situ* analyses of prehistoric pigments, as it is possible to identify their chemical composition without damaging the decorated panels.

### Raman spectroscopy

During the second stage, four samples of black pigments were taken from Les Dogues: three of them from cavity I (DOGUES_1; DOGUES_2; DOGUES 3) and the fourth from cavity II (DOGUES_4) (Fig 3). The sampling was carefully carried out using a sterilized scalpel blade. Materials were immediately stocked in Eppendorf vials to avoid any kind of contamination. Samples (<1mm) were mainly taken from areas of the motifs that were already damaged, yet were still representative of the two Levantine styles identified in Les Dogues. This particular sampling constraint was conducted in accordance with the protection policy governing Spanish Levantine paintings as World Heritage. Samples were prepared in cross sections and embedded in polyester resin to make their manipulation easier.

Initial study of these samples was conducted using a Leica M165 stereomicroscope (magnification range 7.3X and 120X). Subsequent molecular identification of the Les Dogues pigments was performed by means of Raman Spectroscopy. Raman spectra were obtained using a HORIBA Jobin Yvon iHR320 spectrometer with a Peltier-cooled CCD for detection and a 532 nm doubled YAG laser as excitation. A 50x magnification LWD objective was used to focus the laser on the sample and collect the scattered light. The measurements were performed using a laser power of 1.5mW, an integration time of 20 s and up to 36 spectral accumulations to achieve an appropriate signal-to-noise ratio. Wavenumber shift calibration was accomplished with silicon phonon frequencies. Raman spectra were collected from different points within each sample under the same conditions.

### Optical Microscopy (OM) and Scanning Electron Microscopy with Energy Disperse X-ray analysis (SEM-EDX)

The structural and morphological analyses of the Les Dogues black pigments were performed with both a Hitachi S-4800 SEM with a field emission gun (FEG) spotlight and a resolution of 1.4 nm at 1 kV, and a Hitachi S-4100N VP-SEM with an energy dispersive BRUKER XFlash X-ray spectrometer, operating at 15 kV and a working distance of 1 cm. Prior to the SEM-EDX analysis, the samples were metallized with a gold-palladium alloy to improve their conductivity and to optimize the capture of high-resolution images, especially of the pictorial layers.

SEM-EDX microanalysis provided significant data regarding the elemental composition of the samples. This data was subsequently compared to results from the *in situ* EDXRF analysis.

### Archeobotanical analysis

After combustion the anatomical structure of wood remains, thereby permitting the botanical identification of plant species via the analysis of charcoal, while the level of taxonomic identification is a function of the size of samples, the anatomical characteristics of the wood, as well as the degree of preservation. In our case study the archaeobotanical study was restricted to the identification of plant cells due to both poor preservation and the small size of charcoal particles contained in the Les Dogues pigments. Detailed observation and micrography were carried out using a Hitachi S-4100 Field Emission SEM and the EMIP 3.0 (Electron Microscope Image Processing) software package.

### Experimental replication of pigment elaboration

For a better understanding of the data from physicochemical analyses of the prehistoric pigments from Les Dogues, notably regarding the structure and the state of preservation of plant tissues, and in an attempt to reveal the underlying operational sequences of pigment production and use, an experimental protocol for the replication of pigments was developed. The first goal of experimental replication was to create reference samples of pigments resulting from different *chaînes opératoires* and raw material mixtures. A second goal was to make inferences concerning the technical gestures involved in the transformation of charcoal into pigment, as well as the identification and use of possible binders in mixtures. To reach these goals, the structure and degree of fragmentation of vegetal tissues and cells of recreated pigment samples were compared with prehistoric samples by means of SEM.

**Fig 5:**
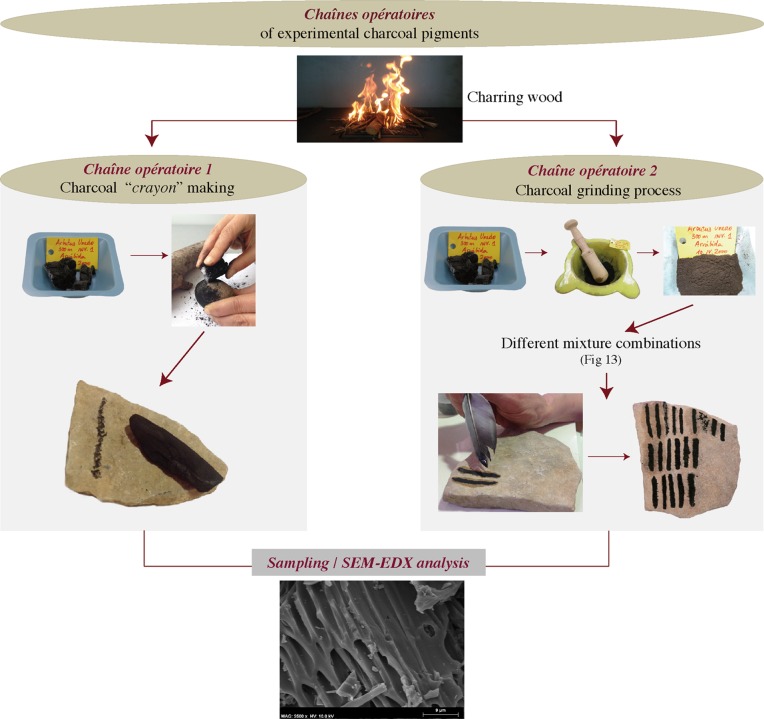
Protocol of the two replicated *chaînes opératoires* that were carried out.

Several criteria were taken into account for the development of this experimental replication (Fig 5). Firstly, the data provided by the physicochemical characterisation of the Les Dogues pigments, and the botanical identification of their charcoal particles, allowed the selection of the principal raw material. Plants considered for the experiment fulfilled the following requirements: their anatomy had the same characteristics as those identified in the Les Dogues pigments, they were abundant in Iberian Neolithic Mediterranean forests, and they were frequently used as fuel or as a raw material in the manufacture of tools by Neolithic societies [24–29]. The first step of the experimentation therefore consisted of the charring of wood from the selected plant species in open-air fires. Secondly, the structure and degree of fragmentation of charcoal particles of the Les Dogues pigments permitted for the proposal of at least two hypothetical *chaînes opératoires* for their elaboration. The second step of the experiment was thusly the replication of these two sequences of technical gestures. The first sequence involved the producing of a charcoal *crayon* by gently sharpening one of the ends of a charcoal fragment by continuously scraping it against limestone (*chaîne opératoire* 1). The second sequence of technical gestures reproduced involved the intense grinding of the charcoal followed by its mixture with solvent and binding agents (*chaîne opératoire* 2.1) or with only solvent agents (*chaîne opératoire* 2.2). 3 g of charred wood of different species were ground for 25–30 minutes with a stone mortar and a wooden pestle. The result was a very fine textured powder. Different mixtures were tested, using water or milk as solvents and honey, egg white, or animal fat as binders. Data from ethnographic studies [30–31], recent research focused on assessing variability in *chaînes opératoires* of pigment production [32–34], physicochemical characterization of recipes and “paint pots” of the prehistoric colour palette [35–37], as well as other experimental approaches to pigments in Palaeolithic art [38], all provide justification for the selection of these substances as solvents or binders. The last step of experimentation involved the use of produced charcoal *crayons* (*chaîne opératoire* 1) and the different pigment mixtures obtained (*chaînes opératoires* 2.1 and 2.2) on a limestone plaque from the Valltorta-Gassulla area. Different lines were drawn directly using the charcoal *crayons*, while a bird's feather was used as a paintbrush to test the mixtures, allowing us to assess the quality and fluidity of each mixture when applied using continuous strokes. After the application of the different pigments/mixtures the plaques were left to dry during a two-month period in a sheltered open-air space. The goal was to both consolidate and improve their adherence to the rock support. Actual simulation of weathering processes for comparison between replicated and prehistoric pigments was not conducted at this point, as it was not necessary for our research purposes. Laboratory controlled exposure of experimental pigments to environmental factors will be explored in the future.

Samples were taken from the different strokes drawn with each of the mixtures and the two charcoal *crayons*. They were analyzed with a LEO 435VP SEM, in the ENCIACET-CIRIMAT of the National Polytechnic Institute of Toulouse (France), and with a Hitachi S-4100 Field Emission SEM, in the SCSIE of the University of Valencia (Spain). The aim was to observe possible transformations in the wood’s anatomy after having been subjected to different mechanical actions.

## Results

### Non-destructive analysis

**Fig 6:**
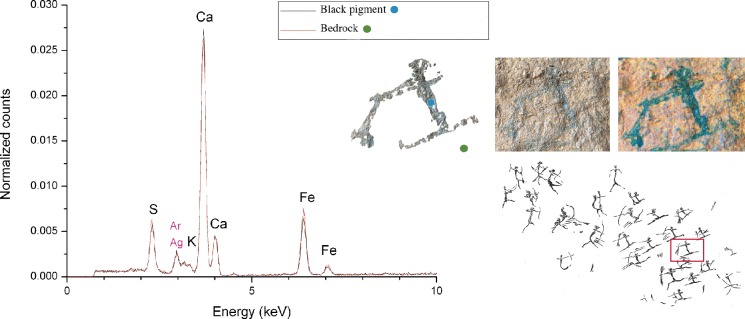
EDXRF Spectra. The black line represents the spectrum of black pigment from one archer involved in the battle scene of cavity I; the red line shows the EDXRF Spectrum of the bedrock. On the right, digital tracing showing the measurement points of the pigment, in blue, and the bedrock, in green; photograph and image modified using colour space transformations (D-Stretch for Image J) to improve the visibility of the motif.

Previous analyses of the rock shelter wall surfaces of the Valltorta-Gassulla region, carried out using XRD and EDXRF, showed the presence of sulphates, calcium carbonates, and iron oxides [18]. This implies the presence of a surface patina, consisting of these aforementioned elements, which covers both the figurative motifs and the limestone surface. Given that the X-rays applied to the area of interest had enough energy to penetrate through both the patinas and the painted layer of the motifs themselves, the discrimination of the characteristic elements of the pigment from those of the rock surface was made via direct comparison of the EDXRF spectra of painted surfaces to those derived from the nearby bare rock. No differences were observed in the elemental composition and in the fluorescence line intensities of the EDXRF spectra from black motifs and those from the rock wall. The EDXRF spectrum corresponding to one of the archers located in cavity I (Fig 6) is representative of all the registered spectra: sulfur (S), calcium (Ca) and iron (Fe) were detected as the main elements both in the paint layer and on the underlying rock. This is turn highlighted that the black pigment must therefore be composed of light elements that cannot be detected by the portable spectrometer, suggesting the use of carbon based pigments and excluding manganese oxides as a possible raw material [20].

### Raman spectroscopy and OM analysis

Clear evidence of the presence of Raman bands belonging to amorphous carbon were found in samples DOGUES_1 and DOGUES_4, whereas morphological structures of charcoal cells were identified in samples DOGUES_2 and DOGUES_3.

**Fig 7:**
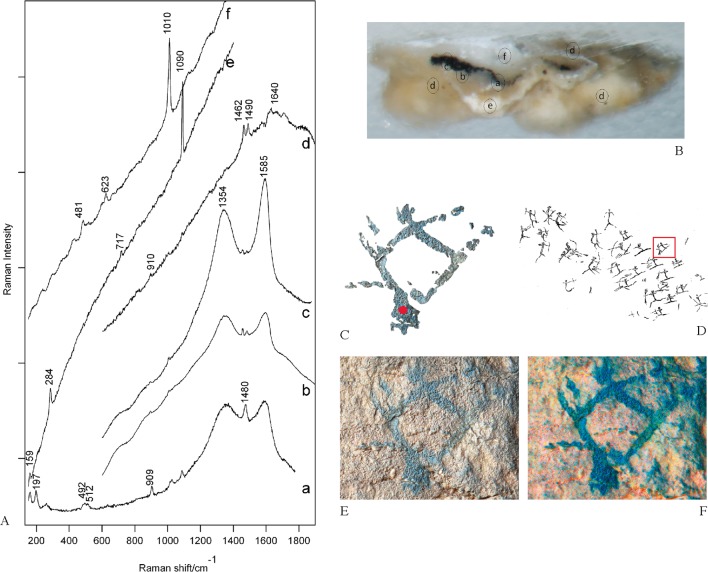
**Raman spectra (A) of representative points of the DOGUES_1 sample from one archer from the battle scene in cavity I (C-F).** B. OM image of a cross-section of the DOGUES_1 sample illustrating its microstratigraphy. Black pigment showing bands of amorphous carbon (a-b-c). Bands of whewellite and weddellite from the substratum (d). Accretion with calcite and gypsum bands (e-f). C. Digital tracing of the sampled archer showing the point of analysis. D. Tracing (by J.B Porcar) of the battle scene showing the position of the sampled archer. Battle scene reprinted from Archivo de Prehistoria Levantina under a CC BY license, with permission from the Editor-in-chief, original copyright 1953. E. Photograph. F. Image enhancement using D-Stretch for Image-J.

The OM of all the samples showed a discontinuous black paint layer located between strata of calcareous compounds; the morphology presented opaque black-coloured particles with variable shapes. Fig 7 shows an OM image of the stratigraphic structure of the sample DOGUES_1, which is representative of the rest.

Raman spectra of DOGUES_1 and DOGUES_4 samples, excited at a wavelength of 532 nm, showed an intense spectral background of fluorescence radiation. It was possible to distinguish whewellite, gypsum, and calcite Raman bands from the intense background of compounds that unambiguously appeared on the cross section of a sample from the decorated panel. The interpretation of the Raman spectra was carried out by comparing the Raman spectra of the Les Dogues samples to reference Raman spectra of the compounds registered in various databases [39] and those from the carbon-based black pigments replicated in our laboratory [20].

Raman spectra of the DOGUES_1 and DOGUES_4 samples were similar. Fig 7A shows Raman spectra of the DOGUES_1 sample, in which the presence of two broad bands at 1354 and 1585 cm^-1^ from amorphous carbon (Fig 7A a-c; Fig 7B a-c) highlights the use of charcoal as the raw material [40–41]. This is also supported by the absence of both the additional band of the phosphate group (νs PO_4_^3-^) in the region of 960–980 cm^-1^ and the band of manganese oxides in the region of 600 cm^-1^. These absences exclude, respectively, the possibility that charred bone or manganese were used as raw materials. Strong bands of whewellite (calcium oxalate monohydrate, CaC_2_O_4_ • H_2_O) at 1462 and 1490 cm^-^1 and weddellite (calcium oxalate dihydrate, CaC_2_O_4_ • (2+x)H_2_O) at 910, 1480 and 1640 cm^-^1 were also observed on the whitish substratum (Fig 7A-d; Fig 7B-d). These oxalates are frequently found in the form of coatings and encrustations on rocks exposed to the atmosphere. There are several hypotheses as to the origin of calcium oxalate coatings, ranging from their being a result of human activity, to the metabolic action of lichens, bacteria, and microbes inhabiting the outer layers of rock faces [4, 42–43]. It is likely that these crusts are the cause of the very strong spectral background of fluorescence radiation that was observed when studying the samples using Raman spectroscopy. Strong bands of gypsum (CaSO_4_ • 2H_2_O) at 1010 cm^-1^ (Fig 7A-f; Fig 7B-f) associated with the symmetric stretching mode of the tetrahedral sulphate anion, as well as the presence of calcite (CaCO_3_) at the 284 and 1090 cm^-1^ bands (Fig 7A-e; Fig 7B-e), were detected in the inner and outer layers of the figurative motifs.

### Archeobotanical study

Having confirmed the presence of charcoal in the DOGUES_1 and DOGUES_4 samples, an exhaustive observation of the DOGUES_2 and DOGUES_3 samples was carried out with SEM-EDX in order to, firstly, taxonomically identify the plants used as raw materials, and secondly, to understand the preparation processes of these pigments by evaluating the degree of fragmentation and size of the plant structures, as well as their state of preservation.

**Fig 8:**
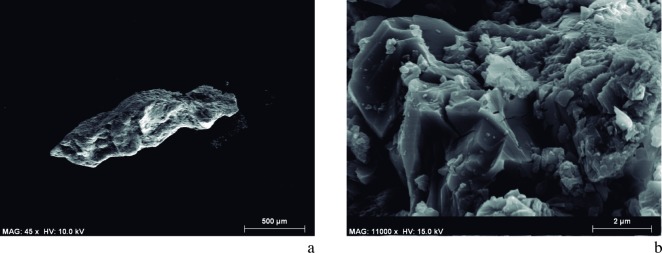
SEM Microphotographs showing the content of the DOGUES_2 and DOGUES_3 samples. a. Panoramic view of the DOGUES_2 sample. b. Disintegrated plant tissue identified in DOGUES_2.

**Fig 9:**
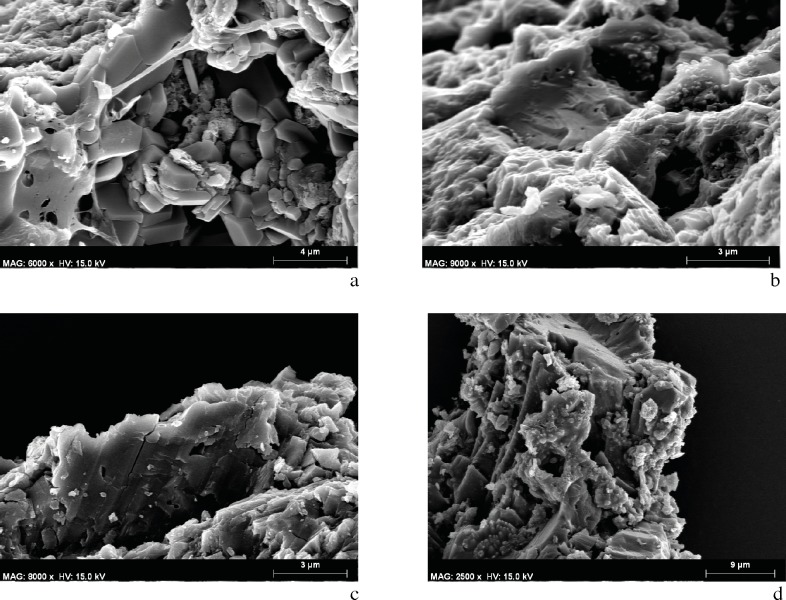
SEM microphotographs of conifer cells in the DOGUES_2 and DOGUES_3 samples. a-b. Fractured cell and possible ray cells identified in DOGUES_2. c-d. Possible tracheid and cells in anatomical connection from DOGUES_3.

**Fig 10:**
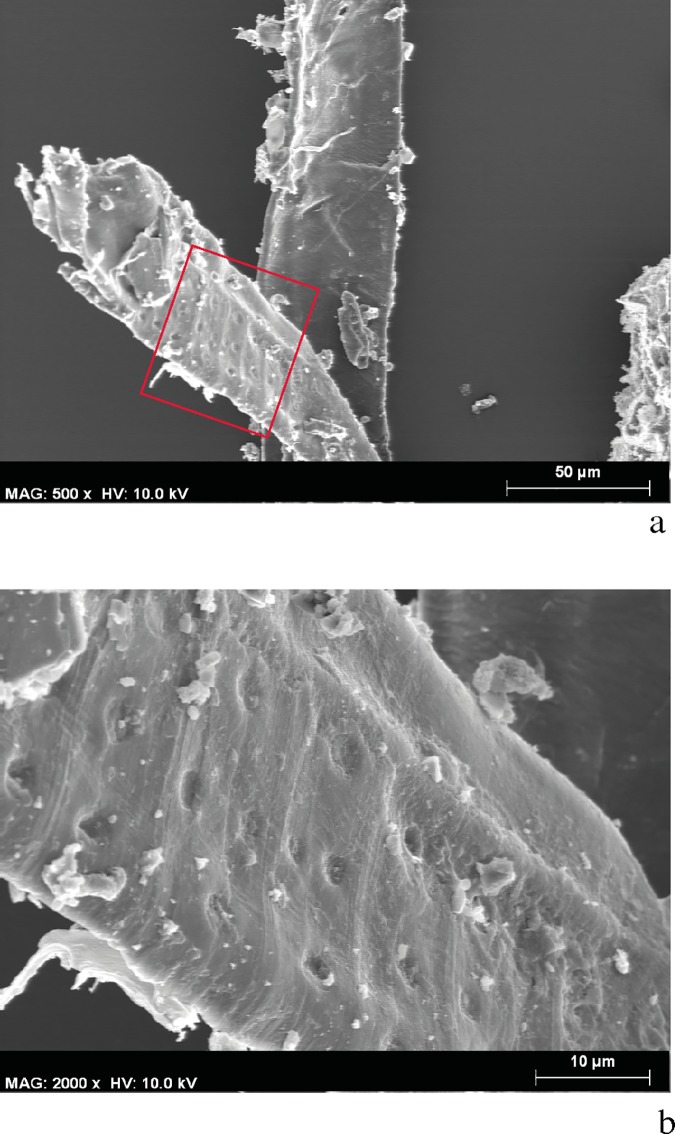
SEM microphotographs of an angiosperm cell from sample DOGUES_2. a. Vessel. b. Detail of spiral thickenings and pits.

The archeobotanical study was rendered difficult due to the small size of the samples (Fig 8A), the disintegration of tissue (Fig 8B), and the incrustation of substances within the cells causing them to seal. SEM microphotographs showed broken, almost disintegrated plant tissues where hardly any diagnostic elements were preserved. The identified plant cells were found in totally isolated positions. Some of them were hardly connected to other cells, but even in those cases the tissues could not be clearly distinguished. Despite all these difficulties, conifer particles were detected in the DOGUES_3 sample based on the identification of both possible tracheids with severely obstructed pits (supposedly areolate), and other cells that could be related with xylem ray cells (Fig 9). In spite of the difficulty we faced when trying to identify anatomical plant structures, an angiosperm cell was also identified in the DOGUES_2 sample, corresponding to the vascular tissue; a vessel (50 μm diameter) with spiral thickenings and large pits was also observed (Fig 10A). In Fig 10B it is possible to see that the pits, which are always collapsed, are aligned perpendicularly to the spiral thickenings, and sit between them. The size of the charcoal particles rarely exceeds 100 μm in length and their morphology is round or angular in shape.

**Fig 11:**
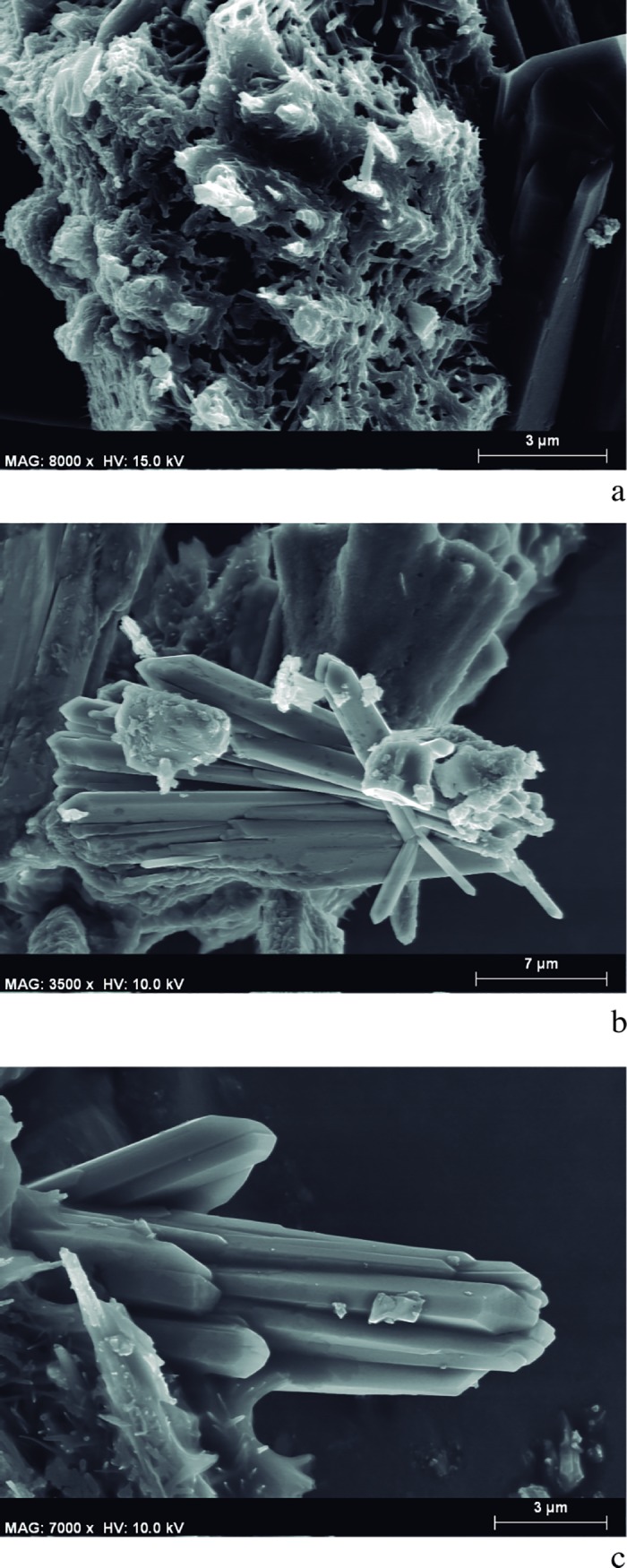
SEM Microphotographs. a. Organic structures (fungal or bacterial microorganisms) in the DOGUES_3 sample. b-c. SEM microphotographs of calcium oxalates of the DOGUES_2 sample.

In addition to plant cells, indefinite organic structures, corresponding probably to fungal or bacterial microorganisms (Fig 11A), and calcium oxalate crystals (Fig 11B and 11C), compounds that may be synthesised through the action of microorganisms from the rock components or from potential binders or organic contents used in the preparation of the pigments [4, 42–43], were also observed.

The type of vessels identified in the Les Dogues samples is an inherent feature of numerous Mediterranean plants, such as the Strawberry tree (*Arbutus unedo*), the Mastick tree (*Pistacia lentiscus* and *Pistacia terebinthus*), Evergreen buckthorn (*Rhamnus alaternus*), Rosaceae (*Prunus)* or woody fabaceae. The observation of a single anatomical element, therefore, did not permit a more specific botanical identification. However, this study has highlighted the use of various species in the preparation of these pigments, since both conifer and angiosperm cells were identified in the DOGUES_2 and DOGUES_3 samples. Additionally, the high fragmentation and the morphology of the charcoal particles, rendering the identification of tissues quite difficult [35–36], suggest that charcoal was subjected to an intense, destructive mechanical process during the preparation of pigments. Furthermore, the poor SEM image definition of the plant cells raised the possibility that the powder resulting from the grinding process of charcoal was mixed with binding and/or solvent substances of a fatty nature. These substances probably penetrated and filled the cells, hiding in part some of the elements of their anatomical structure, thus complicating their identification.

### Replicated pigments analysis

For the experimental replication of charcoal pigments the following plants were selected: among angiosperms, wood from species with spiral thickenings in their cells–Strawberry tree (*Arbutus unedo*), Mastick tree (*Pistacia terebinthus*), Black buckthorn (*Rhamnus lycioides*) and Mock privet (*Phillyrea latifolia*)–; among conifers, wood of the Aleppo pine (*Pinus halepensis*). The SEM analysis of samples from replicated pigments made it possible to both observe the transformation undergone by plant tissues and to characterize the changes seen in the wood’s anatomical structures in the two experimental *chaînes opératoires*.

#### *Chaîne opératoire* 1

**Fig 12:**
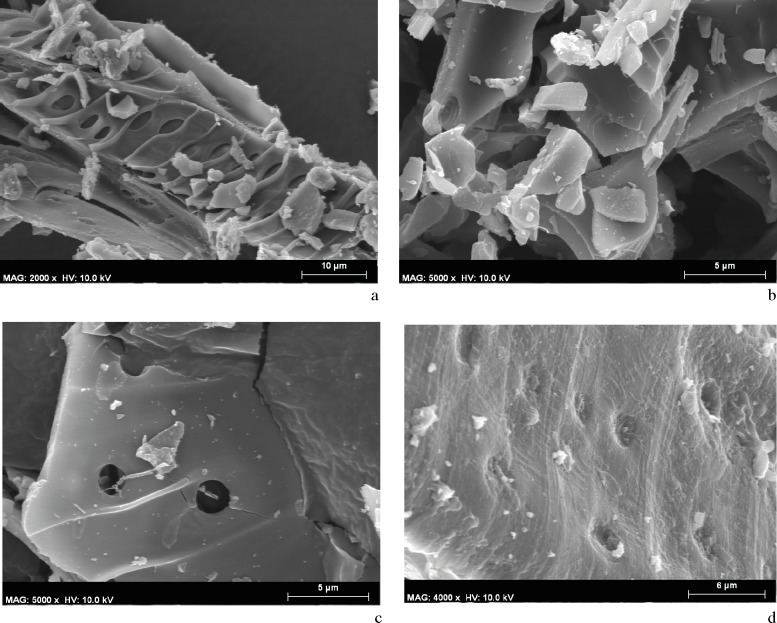
SEM Microphotographs. a. Sample taken from a stroke drawn with an experimental *crayon* made from *Arbutus unedo* charcoal. Plant tissues are anatomically connected and a precise botanical identification is possible. b-c. Sample taken from a stroke drawn with an experimental *crayon* made from *Pistacia terebinthus* charcoal. Small sized particles can be also observed, but pits and spiral thickenings are not obstructed. d. The DOGUES_2 sample, showing obstructed pits and spiral thickenings that have almost disappeared.

Two *crayons* were made with charcoal from *Arbutus unedo* and *Pistacia terebinthus*. Samples from the strokes drawn with each of these two *crayons* showed particles larger (≥1000 μm) than both those in *chaîne opératoire* 2 (grinding and mixture) and in the pigments of Les Dogues. The plant tissues were anatomically connected and were easily recognizable, thus facilitating the identification of diagnostic anatomical elements from each taxon (Fig 12A). Some small sized particles (≤ 20 μm), for which it was not possible to identify the taxon, and even smaller particles corresponding to unspecific plant cells were also observed (Fig 12B). At any rate, the cells were clean and the pits and spiral thickenings were not obstructed making them clearly observable (Fig 12C).

The characteristics of the replicated pigments from this *chaîne opératoire*–particle size and state of tissue preservation–did not match those from the prehistoric pigments of Les Dogues (Fig 12D). This means that we can safely reject the use of a charcoal *crayon* as a valid hypothesis. The form of the human figures of Les Dogues, small in size and designed with precise linear brush strokes, hardly exceeding 1 mm, supports the rejection of charcoal *crayon* use and underscores the likely application of a fluid pigment with a fine brush. Moreover, given that these paintings are exposed to the open air, the use of some sort of substance that augments the adherence of the pigment to the rock seems imperative.

#### *Chaîne opératoire* 2

**Fig 13:**
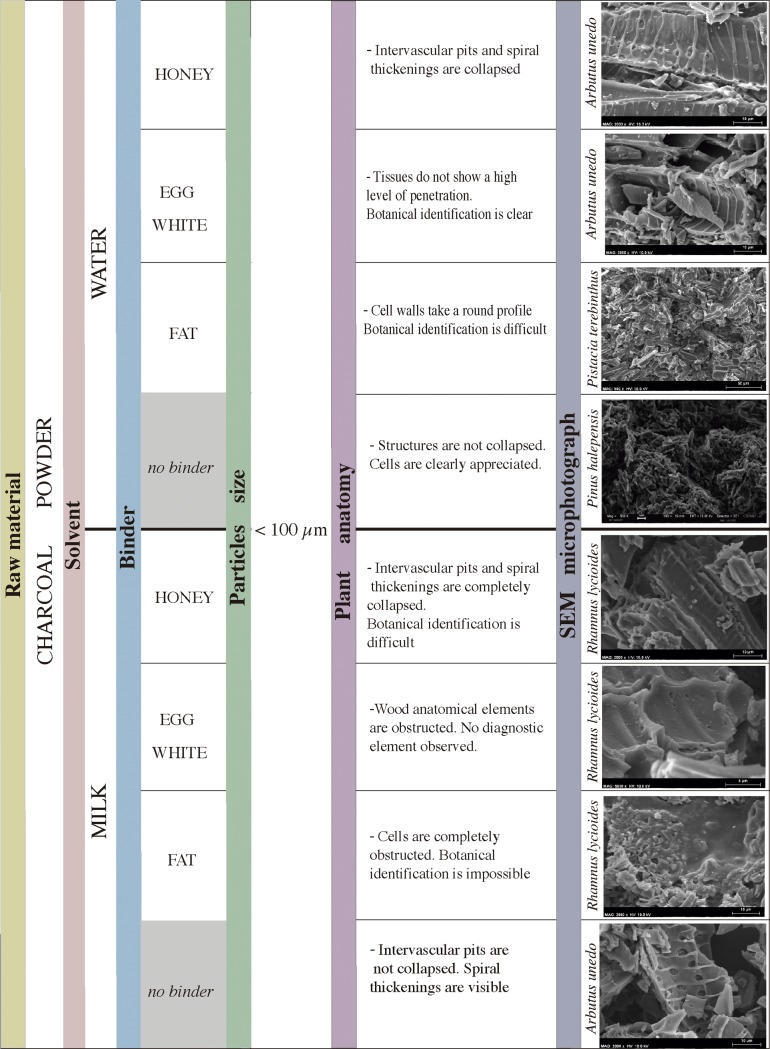
Experimental replication of *chaîne opératoire* 2. Different raw materials and binding mixtures carried out. SEM Microphotographs of different samples taken from the replicated strokes.

**Fig 14:**
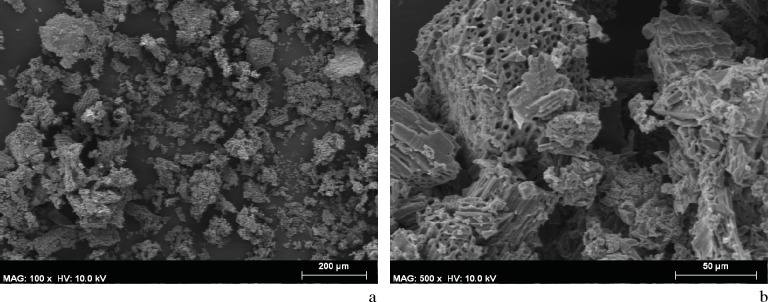
SEM microphotographs. a-b. Sample taken from a stroke drawn with a pigment prepared through a mixture of *Rhamnus lycioides* charcoal powder, milk, and egg white.

The results from the different combinations of mixtures tested are summarised in Fig 13. With regard to the size of particles, the anatomical structures of the wood were severely damaged and fractured in all the mixtures tested (Fig 14A). The average size of the particles is below 100 μm, rendering their botanical identification difficult. As an exception, some larger particles (≥ 100 μm) were observed, but even in these cases the identification of tissues was only possible because the plant matter used was already known (Fig 14B); in a blind study we would not be able to identify particles to the genus or species level.

#### *Chaîne opératoire* 2.1

**Fig 15:**
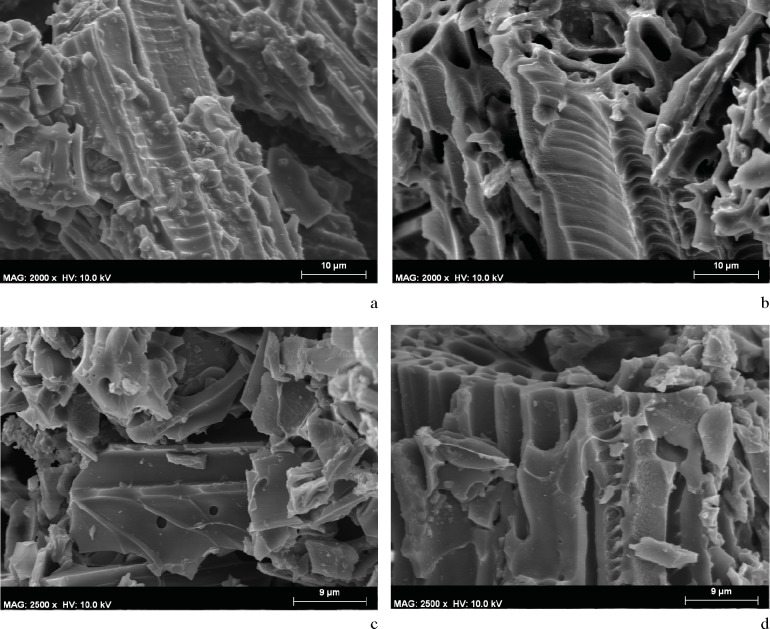
SEM microphotographs. a. Sample from the pigment prepared with a mixture of *Rhamnus lycioides* charcoal powder, water, and honey. b. Sample from the pigment prepared with a mixture of *Arbutus unedo* charcoal powder, water, and fat. c. Sample from pigment prepared with a mixture of *Pistacia terebinthus* charcoal powder, water, and egg white. d. Sample from pigment prepared with a mixture of *Rhamnus lycioides* charcoal powder, milk, and egg white.

The use of water or milk as solvents in the different mixtures did not produce significantly different results. The combination of either water or milk with honey resulted in charcoal particles being both firmly bonded together and to the limestone medium. The fluids completely penetrated the cells, masking the intervascular pits and spiral thickenings in both small and even larger particles measuring more than 100 μm (Fig 15A).

Another mixture that penetrated easily, obstructing the cells, included animal fat as a binder. In this case, and regardless of whether water or milk was used as a solvent, the plant particles were highly dispersed, the anatomical elements were completely obstructed, and it was almost impossible to recognize the cells, whose walls had taken on a rounded profile. Botanical identification was difficult even with particles over 100 μm (Fig 15B).

Recipes that used egg white as a binder showed different behaviours in the plant tissues, depending on whether the solvent was water or milk. In the first case the tissues did not show a high level of penetration, meaning cells remained clearly visible (Fig 15C) and particles larger than 100 μm were easily identifiable. In the second case the wood’s anatomical elements were obstructed by the different binding ingredients used, hindering the observation of the diagnostic elements (Fig 15D).

#### *Chaîne opératoire* 2.2

**Fig 16:**
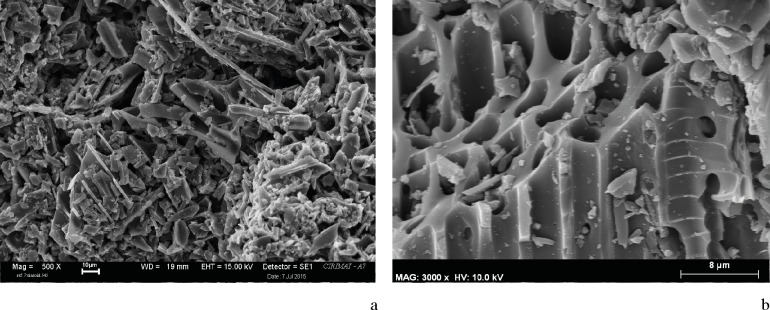
SEM microphotographs. a. Sample from the pigment prepared with a mixture of *Pinus halepensis* charcoal powder and water. b. Sample from the pigment prepared with a mixture of *Rhamnus lycioides* charcoal powder and water.

Regardless of the plant species used, charcoal particles were highly dispersed and were easily detachable from the limestone medium (Fig 16A). The solvent penetrated the anatomical structures but did not cause them to collapse, meaning the tissue could be clearly observed and even the relief of the angiosperm spiral thickenings could be distinguished (Fig 16B).

### A comparative analysis of prehistoric and replicated pigments samples

Comparing the data from the replicated pigments with those from the DOGUES_2 and DOGUES_3 samples we can conclude that:

- After an intense experimental grinding process, the plant tissue of replicated pigments was severely damaged. Nevertheless, particles larger than 100 μm were still preserved, enabling the identification of cells. In the samples of Les Dogues, very few particles are over 100 μm, leading us to suppose that the grinding process was perhaps longer/more intense.
  ○ The dissolution of the charcoal powder in water or milk (*chaîne opératoire* 2.2) did not obstruct the tissue and made it possible to clearly discern the diagnostic elements of the plants that were used.
  ○ The incorporation of binders (*chaîne opératoire* 2.1) generated diverse results. While animal fat and honey, dissolved in water or milk, had a high capacity to obstruct cells and made the identification of anatomical features difficult, egg white did not seem to have the same capacity to penetrate and obstruct, particularly when it was dissolved in water. Therefore, its presence did not prevent the anatomical analysis of charcoal particles.
  ○ Considering these experimental results and the state of preservation of the angiosperm identified in the DOGUES_2 sample, we can say with a reasonable degree of certainty that both the intense grinding of charcoal and the use of viscous binding agents played major roles in the preparation process of the prehistoric pigments used at Les Dogues. These binding agents could include animal fat or honey, which obstructed cells and consequently prevented the clear identification of spiral thickenings and pits in the vessels. From a strictly technical standpoint, pigment quality during application seemed constant regardless of whether recipes used fat or honey, yet the latter substance did create a colourless halo as a result of its penetration into the porous limestone. This could imply that pure honey was not used as a binder in prehistoric examples as they do not present the same colourless halo, but only if post-depositional processes are not responsible for its disappearance.

## Discussion

The project carried out at Les Dogues has shed new light on the composition and preparation of black pigments in Spanish Levantine rock art. This research presents a number of new aspects. From a methodological standpoint, the characterization of prehistoric pigments by crossing different techniques of analysis (EDXRF, Raman, OM, SEM-EDX and Anthracology) with experimental archaeological protocols constitutes a novel approach to Spanish Levantine paintings. This new approach, elaborated within the *chaîne opératoire* theoretical and methodological framework, allows for a comprehensive exploration of social identity through the study of the different stages of pigment processing, as these represent particular learned traditions of pigment elaboration. The characterization of recurrent *chaînes opératoires*, therefore, is extremely relevant for the definition of prehistoric societies and their cultural boundaries from a synchronic perspective, whereas diachronic variability in these traditions within and between chronological phases could highlight significant social and technological evolution.

Concerning the first step of the *chaîne opératoire*–the selection and procurement of raw materials–the EDXRF analyses firstly identified the use of black carbon-based pigments, while subsequent Raman analyses confirmed the use of charcoal through the identification of charcoal-corresponding bands. Observation of samples using OM and SEM enabled the further botanical identification of the charcoal, corresponding to two major types of plants: angiosperms and conifers. The poor preservation of charcoal particles however rendered identification to the species level impossible.

Angiosperms with spiral thickenings in their vessels were abundant in Iberian Mediterranean Neolithic forests and were frequently used as fuel. The archaeobotanical data from the Neolithic site of Cova Fosca (Ares del Maestre, Castellón) [24], in the immediate surroundings of Les Dogues, have provided charred plant remains such as angiosperms with spiral thickenings in their vascular vessels or fibres (*Prunus* and cf. *Pistacia lentiscus*), and conifers (*Juniperus* and *Pinus sylvestris or Pinus nigra*), similar to those detected in the samples of Les Dogues. However, the alteration of tissues observed in the DOGUES_2 and DOGUES_3 samples, as well as the identification of just one diagnostic element, impeded a direct association between the charcoal used with the specific species documented in the Gassulla area.

Regarding raw material procurement, at this stage in our research it is difficult to determine whether specific plant species were collected explicitly or whether charcoal from domestic hearths was opportunistically recycled for secondary use in pigment preparation. As of right now we still lack a representative sample of Levantine charcoal pigment; a single stylistically homogenous scene, likely produced at a single moment in time with a single “paint pot”, has been analyzed using the above-proposed methodology, meaning we cannot push interpretations further for the time being.

With regard to the second and third steps of the *chaîne opératoire*–preparation process and application–the small size of the particles, the anatomical disconnection, and the saturation of the cells observed in the samples of Les Dogues underline a complex processing of charred plant matter, and concomitantly reject the hypothesis of direct use of a charcoal *crayon*. The experimental replication of two different *chaînes opératoires* and the comparative SEM analyses of prehistoric and experimental samples has allowed for a greater understanding of tissue alteration identified in prehistoric pigments. An intense grinding of the charcoal would explain the small size of particles, as well as the generally poor preservation of the vegetal anatomy, while the use of a dense, fatty binder could be responsible for the infilling of the identified plant cells. This calls into question the prior assumption that Levantine paintings were the product of a simple solution and not complex mixtures [7]. Although the lack of spectra corresponding to organic binders is a constant in the IR and Raman analyses of Levantine pigments [44, 20], the results of our research demonstrate a more complex elaboration process in which binding substances likely played an important role. These substances improve the consistency of mixtures and augment the adherence of pigments to the limestone medium, yet their traces may have disappeared or been weakened as a result of intense bacterial activity, already known to have been a factor in the general degradation of the paintings under study. This hypothesis must be verified with other more precise techniques, such as gas chromatography (GC), which has already provided promising results in other studies of Palaeolithic pigments, such as the detection of animal and plant fats as binders [45–47].

The *chaîne opératoire* described above, which corresponds to a sequence of technical gestures and contextually embedded decision-making processes that accompany the life-history of raw material, from acquisition to final product, as already underlined, reveals the interplay between socially rooted learned traditions and motor habit acquisition, and can therefore be considered as a reflection of the social identity of the authors [13]. This innovative approach to Levantine paintings, combined with the formal analysis of motifs at different geographical scales, will allow for a better characterization of stylistic horizons and their territorial boundaries, as well as for a diachronic assessment of the horizontal transmission of techniques.

**Fig 17:**
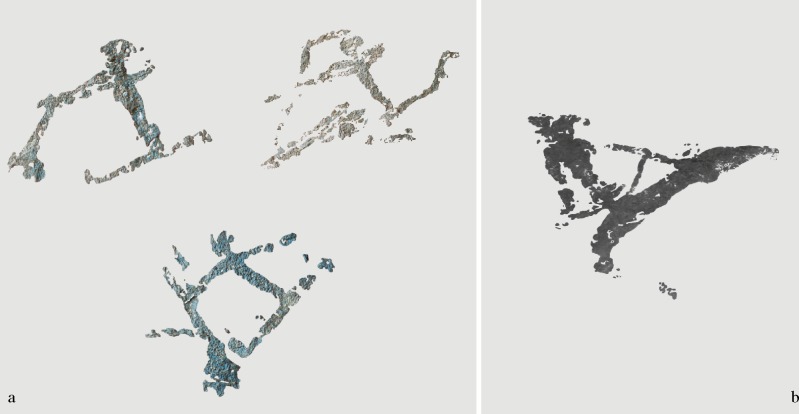
Digital tracings of human motifs sampled from Les Dogues. a. Linear motifs located in cavity I. b. Archer located in cavity II.

Our research in Gassulla, currently focused on three rock art shelters, indicates that the use of black colour is quantitatively more common than expected in this area: up to 48 figures in Cova Remigia [20], 1 in Cingle de la Mola Remigia [22] and 34 in Les Dogues. All these black figures were depicted using charcoal pigment; as of yet, the presence of manganese as a main component or as an extender in charcoal pigment has not been detected. This mixture of organic and mineral components has been identified in some black Palaeolithic paintings, possibly as a way to augment the intensity of the black colour [48]. It must be stressed that the actual number of figures drawn with charcoal pigment in Gassulla was likely more important. This type of pigment is not very stable and has a remarkable sensibility to light. These combined with other weathering factors likely compromised the long-term conservation of figures [17]. Additionally, the identification of motifs, even using IR images or colour enhancement filters, is arduous.

Les Dogues has provided relevant new data concerning Levantine black pigments: on the one hand, it is the only site in which charcoal pigment was exclusively used; on the other hand, the formal differences between the motifs portrayed allow us to advance new arguments concerning the temporal and cultural patterns of this kind of pigments. To date, all figures drawn with charcoal pigment in the Gassulla area share stylistic features, such as an extremely formal simplification of human anatomy, small size, and a remarkable dynamism in the compositions, representing hunting or violent scenes [20; 22]. These formal attributes are distinctive of the last stylistic phases–the Linear horizon–of the regional sequence [49–51]. The formal features of human motifs documented in Les Dogues, however, argue for the presence of two different stylistic phases. The human figures in the battle scene of cavity I would stylistically fit into this Linear horizon: small size, anatomical simplification, body disproportion, and extreme dynamism (Fig 17A); while the archers of cavity II, on the other hand, do not share this formal simplification, showing a more naturalistic and well-proportioned anatomy, typical of earlier stylistic phases (Fig 17B).

Bearing these formal differences in mind, we can attribute the use of charcoal pigments in the Gassulla area to two different stylistic phases. Furthermore, it is worth noting that the Linear horizon also shows a wide range of formal variation and an extensive geographic distribution in all Levantine regions, all of which points to a long period of use of this type of figures. Even if we cannot define the specific time lapse characterized by the use of charcoal pigments, due to the lack of radiocarbon dates, the fact that they were used in two different stylistic phases raises new questions with regard to the mechanisms responsible for the transfer of knowledge and know-how of pigment production within Spanish Levantine rock art traditions. Additionally, Raman spectra did not highlight any differences in the samples taken from the two motifs belonging to these two different styles.

Another aspect yet to be explored is the geographical distribution of Levantine charcoal pigment use. To date, outside the Gassulla area, amorphous carbon has only been detected in black pigments of the Ciervos Negros rock art shelter (Moratalla, Murcia) [52], about 350 km away. It must however be stressed that there is an important knowledge-gap concerning black pigments composition in other Levantine regions. To begin to understand the geographical organization of charcoal pigment use we need to approach the physicochemical characterization systematically in regions neighbouring the Gassulla area. This expansion of the scope of analyses is the only way to distinguish between hypotheses of regional particularities or inter-regional diffusion: was charcoal pigment use a regional phenomenon associated with several specific stylistic phases, or does its appearance instead represent regional and macro-regional interaction networks? These further analyses will allow us to explore differential patterns of charcoal or manganese pigment use, the relationship between black and red pigments in the area, and whether these correspond to specific culturally conditioned choices, limited access to raw materials, or other, to date undefined, factors.

It is also worth noting that the identification of organic matter in the pigments would make radiocarbon dating possible, potentially enabling us to refine the chronological and cultural context of Levantine rock art. The significant quantity of charcoal currently needed to obtain calibrated ^14^C dates, with reasonable standard deviations, is damaging to the paintings. This means that for the time being, the application of radiocarbon dating methods to the Levantine charcoal pigments is unfeasible.

## Conclusion and perspectives

The study of pigments from Les Dogues has successfully demonstrated the benefits of a multi-analytical approach integrated within the theoretical and methodological framework of *chaîne opératoire*. Such an approach has allowed for the accurate physicochemical characterization of pigment components, in addition to the holistic comprehension of the technical gestures and decision-making processes embedded in the specific techniques of pigment production. Moreover, the botanical identification of plants used in the preparation of these black pigments constitutes a new line of research that will contribute to our understanding of plant resource exploitation by Neolithic communities.

In this study, we have demonstrated the use of binders by comparing plant tissues from both experimentally produced and prehistoric pigments. The next logical step of this research axis will be to investigate binder use in Levantine rock art and to characterize them via the novel application of analytical techniques in this field of study. This will allow for the identification of other organic compounds, which would be a major advance in our comprehension of the elaboration of Levantine pigments.

Moving forward we aim to integrate this new data regarding Levantine pigments, obtained via the experimental reconstruction of their *chaîne opératoire*, into a larger and more complex archaeological discussion regarding the characterization of different Levantine art styles, their areas of influence, and their interaction and exchange networks. All these aspects are integral to the comprehension of the Neolithic expansion into the Iberian Peninsula, whose resulting cultural mosaic requires precise definitions should we hope to apprehend the historical and societal processes in play.
